# A proprietary blend of *Sphaeranthus indicus* flower head and *Mangifera indica* bark extracts increases muscle strength and enhances endurance in young male volunteers: a randomized, double-blinded, placebo-controlled trial

**DOI:** 10.29219/fnr.v67.8972

**Published:** 2023-01-27

**Authors:** Meher Prasanna Rokkam, Olos Gora, Manikyeswara Rao Konda, Ajay Koushik

**Affiliations:** 1 Department of Orthopedics, Meher Hospital, Vijayawada, India;; 2 Department of Physiotherapy, Vijaya Institute Medical Sciences College of Physiotherapy, NTR University of Health Sciences, Vijayawada, India;; 3 Department of Ayurvedic Medicine, Suraksha Health Village, Vijayawada, India;; 4 Department of Orthopedics, Mysore Medical College & Research Institute, Mysuru, India

**Keywords:** *1-RM strength*, *ergogenic herbal composition*, *LI12542F6*, Mangifera indica, Sphaeranthus indicus

## Abstract

**Background:**

The demand for safe and efficacious botanical formulations to increase muscle mass, strength, and stamina is increasing among athletes and the general population. The nutraceutical supplements of medicinal plant origin exert minimal health concern.

**Objective:**

This randomized, double-blind, placebo-controlled study was aimed to evaluate the ergogenic potential of a proprietary, standardized formulation (LI12542F6) of *Sphaeranthus indicus* flower head and *Mangifera indica* stem bark extracts.

**Methods:**

Forty male participants 18–40 years of age were assigned to receive either a placebo (*n* = 20) or 650 mg/day LI12542F6 (*n* = 20) for 56 days. All participants performed a fixed set of resistance exercises during the intervention. The primary endpoint was the change from baseline muscle strength, assessed by one-repetition maximum (1-RM) bench and leg presses, and handgrip strength. The secondary endpoints included cable pull-down repetitions, time to exhaustion on a treadmill, mid-upper arm circumference (MUAC), body composition using dual-energy x-ray absorptiometry (DEXA), and free testosterone and cortisol levels in serum.

**Results:**

Fifty-six days supplementation of LI12542F6 significantly improved baseline bench press (*P* < 0.0001), leg press (*P* < 0.0001), handgrip strength (*P* < 0.0006), number of repetitions (*P* < 0.0001), and time to exhaustion (*P* < 0.0008), compared to placebo. Post-trial, the LI12542F6 group also showed significantly increased MUAC and improved body composition and serum hormone levels. The participants’ hematology, clinical chemistry, and vital signs were within the normal range. No adverse events were observed.

**Conclusion:**

This study demonstrates that LI12542F6 supplementation significantly increases muscle strength and size and improves endurance in healthy men. Also, LI12542F6 is well-tolerated by the participants.

## Popular scientific summary

- The demand for botanical supplements is growing among athletes and non-athletes to increase muscle mass, endurance, and strength.
- LI12542F6 is a standardized blend of *Sphaeranthus indicus* flower head and *Mangifera indica* stem bark extracts.
- Daily consumption of 650 mg LI12542F6 over 56 consecutive days significantly increased muscle strength and size and improved endurance in young male volunteers.
- LI12542F6 is well-tolerated by the participants.

Muscle mass, strength, and endurance are essential for athletic performance and overall health in the general population (1, 2). Most sports nutrition supplements consist of amino acid(s) and/or protein, which support increased muscle mass and strength when combined with a resistance training program (3). Increased physical activities, including resistance training, increase the need for protein consumption. However, excessive protein supplementation offers limited additional benefits (4). The range of available dietary interventions outside the domain of essential nutrients to increase muscle mass, strength, and endurance is limited. Nitrate and caffeine have been shown to increase muscle strength and endurance in the short term (5–7). β-Hydroxy-β-methylbutyrate (HMB), a leucine metabolite, has also been reported to improve several parameters of muscle performance (8, 9).

To develop a scientifically supported, natural product for enhanced muscle performance, we examined a series of botanical extracts for their ability to modulate critical processes in muscle metabolism. Our unpublished *in vitro* experiments evaluated the activation of the Mammalian Target of Rapamycin (mTOR), upregulation of muscle-specific transcription factors, free radical scavenging activity, and enhanced nitric oxide (NO)-generation in a variety of cellular models. The extracts of *Sphaeranthus indicus* flower head and *Mangifera indica* (mango tree) bark exhibited consistent efficacies across the screening assays. Furthermore, these individual extracts showed apparent synergy, especially in enhancing endothelial nitric oxide synthase (eNOS) activity in the endothelial cells (unpublished observations). Physical exercise increases NO synthesis through eNOS activation, and NO improves mitochondrial function via PGC1α overexpression (10, 11). Improved mitochondrial function is essential for endurance exercise adaptation of muscles and sustained performance (12).

*S. indicus* has a long history of usage in Indian traditional medicine, Ayurveda. Its reported effects include immune modulation, hepato-protection, analgesic, antidiabetic, antioxidant, anxiolytic, anti-inflammatory, and antihyperlipidemic activities (13, 14). The whole plant, flowers, seeds, and roots are used in various Ayurvedic preparations (15).

*M. indica* is native to India but is cultivated worldwide in tropical climates (16). The glucosyl xanthone, mangiferin, is found in the bark and leaves of *M. indica* (17). Mangiferin from mango tree bark has been reported to possess antioxidant, antidiabetic, immunomodulatory, anti-genotoxic, and anti-inflammatory properties (18).

Here, we present a double-blind, placebo-controlled human trial conducted on young males to assess the effects of LI12542F6 supplementation in combination with resistance training on muscle strength and endurance. Furthermore, we evaluated the clinical chemistry markers and vital signs and monitored the adverse events (AEs) reported by the study participants.

## Materials and methods

### Investigational product

This proprietary botanical formulation, LI12542F6 (MyoTOR® or RipFACTOR®), contains 65% (w/w) blend of *S. indicus* flower heads and *M. indica* stem bark extracts at a 2:1 ratio, combined with 35% (w/w) neutral excipients comprising a mixture of microcrystalline cellulose powder (MCCP) and Syloid 244FP. The final product was standardized to contain not less than 4% of 7-hydroxyfrullanolide and 2.5% of mangiferin, the phytochemical reference markers of *S. indicus* and *M. indica*, respectively (19). *S. indicus* flower head and *M. indica* bark raw materials were collected from wild-crafted and plantation sources, respectively, in Krishna district, Andhra Pradesh, India. The raw materials were identified by a certified taxonomist and compared with the authentic raw materials. The voucher specimens (#6578 for *S. indicus* and #6246 for *M. indica*) are preserved in the Taxonomy Division of Laila Nutraceuticals R&D Center, Vijayawada, India. Preparation details and analytical procedures of LI12542F6 are described by Nestmann et al. (19). The study sponsor, Laila Nutraceuticals, Vijayawada, India, provided the test material, an authorized certificate of analysis, Material Safety Data Sheet (MSDS), and specification sheets. The final product’s heavy metals, pesticides, and residual solvents were within acceptable limits.

### Clinical study

#### Ethics approval and registration

The present randomized, double-blind, placebo-controlled clinical trial was approved (ECR/563/Inst/AP/2014; Apr 27, 2016) by the Institutional Ethics Committee of the Alluri Sitarama Raju (ASR) Academy of Medical Sciences and was conducted following the International Conference on Harmonization-Good Clinical Practices (ICH-GCP) guidelines and registered with the Clinical Trial Registry of India (CTRI/2016/05/006950).

#### Study participants

Healthy, recreationally active males (*n* = 40), 18–40 years old, were recruited from a local fitness center in Vijayawada, Andhra Pradesh, India. The subjects were familiar with weight training and had at least 6 months of experience in the gym (defined as four times per week for a minimum of 3–4 h per week). All subjects were healthy based on their medical history, vital signs, and routine clinical laboratory examinations. The subjects were recruited for this study based on the inclusion–exclusion criteria (Table 1). The participants were advised not to take ergogenic dietary supplements. Also, they were instructed to maintain their usual diet and adhere to the study training regimen throughout this study. All selected subjects learned the experimental protocol and the trial requirements from the study coordinator. Subjects were informed of potential conflicts of interest. They gave their written consent for participation in the study in compliance with ICH-GCP guidelines. All participants maintained a daily diary, routinely reviewed and endorsed by the study coordinator. The resistance training and evaluation of exercise performances were conducted under the supervision of trained and certified personnel.

**Table 1:** Inclusion–exclusion criteria

Inclusion criteria	Male subjects between 18 and 40 years of age. Subjects familiar with weight training and have at least 6 months of experience with four times per week for at least 3–4 h, weekly (recreational athletes). Agreed to participate in an exercise program (4 days a week, through the study) as per protocol, under the guidance of the physical instructor of the study. Subjects agreed not to consume any ergogenic supplement during this study. Willing to give written informed consent and comply with the trial protocol.
Exclusion criteria	History of use of anabolic drugs, including corticosteroids (e.g. prednisone) and testosterone replacement therapy. Subjects had a history of cardiovascular diseases or respiratory disorders or fasting blood glucose above 125 mg/dL. Presence of thyroid dysfunction, abnormal liver, or kidney functions. Abnormal hematology parameters and HIV positive. Alcohol consumption of >2 standard drinks per day. History of psychiatric disorder. Subjects participated in any clinical study within the past 30 days of the screening visit.

#### Randomization and study plan

**Fig. 1:**
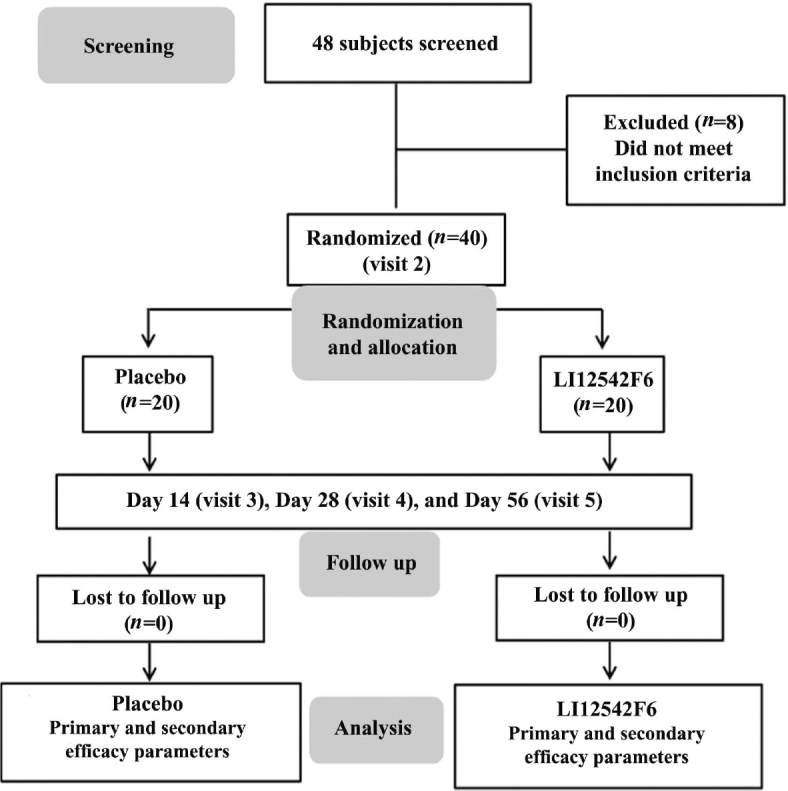
A CONSORT diagram presents the participant enrollment and the following steps of the study.

Forty participants were randomized to receive either 650 mg/day of LI12542F6 or a matched placebo. Randomization code was generated by SAS 9.4 by block randomization using SAS procedure PROC PLAN. Participants were instructed to take either placebo or LI12542F6 capsules every morning for 56 consecutive days. This study included five visits: visit 1 (screening), visit 2 (randomization/baseline), visit 3 (day 14), visit 4 (day 28), and visit 5 (day 56). The coded bottles containing placebo or active capsules were distributed to the participants on the randomization day and days 14 and 28. The supplementation compliance was monitored by counting the unused capsules in the empty bottles on days 14, 28, and 56. The study coordinator routinely confirmed participant adherence to all study instructions. AEs or side effects were evaluated and recorded at all visits. Figure 1 provides a Consolidated Standards of Reporting Trials (CONSORT) flow diagram detailing participant disposition.

The primary efficacy outcome was improved muscular strength from baseline, evaluated by measuring 1-RM (one repetition maximum) in bench and leg press exercises, and handgrip strength. The secondary outcome measures included muscular endurance, measured by the total number of repetitions in a cable pull-down exercise and the time to exhaust in a treadmill exercise. Changes in body composition measured using Dual-energy X-ray absorptiometry (DEXA) and mid-upper arm circumference (MUAC) were secondary efficacy variables. Measurements of serum, free testosterone (free-T), dihydrotestosterone (DHT), cortisol, creatine kinase (CK), and lactate dehydrogenase (LDH) were assessed.

At each follow-up visit, safety and tolerability were monitored by recording vital signs, and AEs were observed by completing daily compliance cards and daily diaries that the investigators reviewed.

#### Resistance training

Subjects followed a training regimen of 4 workouts per week throughout this study. The resistance exercise program included 17 chest/shoulder, back, leg, and arm exercises. The participants performed exercises targeting particular muscle groups each day: chest/shoulder (incline dumbbell press, flat barbell press, dumbbell shoulder press, and lateral raise); back (bent rows, cable pull-downs, one arm dumbbell, and seated rear deltoid raises); legs (calf raises, leg extension, lying leg curls, and leg press); and arms (EZ-bar curl, alternating dumbbell curls, overhead dumbbell extensions, hammer curls, and kickback/cable push down). During the training sessions, participants performed warm-up exercises at 50% of 1-RM in two sets of 8–10 repetitions. Subsequently, the subjects performed 2–3 sets of 10 repetitions at 70% of baseline 1-RM. The participants took 2 min rest between two sets of a particular exercise. Then, the resistance was gradually increased to 90% of the baseline 1-RM. Between any two exercises, participants rested for 10 min. The training programs and performance assessments were conducted under the supervision of trained instructors and study personnel.

On the assessment visits of the study (baseline, and days 14, 28, and 56), only the efficacy assessment exercises—bench press, leg press, cable pull-down, handgrip strength, and treadmill test—were performed.

## Outcome measures

### 1-RM strength

According to the National Strength and Conditioning Association, the 1-RM strength of bench and leg presses was assessed (20). Participants warmed up by performing two sets of 8–10 repetitions at approximately 50% of their anticipated maximum. The subjects then performed successive lifts, starting at approximately 70% of their anticipated 1-RM and increasing by 5 kg until they reached 1-RM.

#### Handgrip strength

A pre-calibrated analog grip dynamometer (Takei Scientific Instruments Co., Ltd, Tokyo, Japan) was used to measure the handgrip strength of their dominant hands according to the standard protocol (21). The participants in standing position with shoulders adducted, arms by their sides with full elbow extension, performed this test. The participants were asked to squeeze the dynamometer with the maximum force for 3 s. Among three consecutive trials, the highest value of strength was recorded.

### Cable pull-down

Muscle endurance was measured as the number of repetitions subjects could complete at 80% of their 1-RM by cable pull-down using a standard lateral pull-down cable system (GML83 Pro Lat Machine, Body-Solid Inc, Forest Park, IL). Using a standard lateral pull-down bar, the participants performed wide grip anterior pull-down exercises with their pronated handgrips. Each pull was conducted from completely extended arms to the bar contact with the chest position (22).

### Time to exhaustion

Participants warmed up for 10 min before beginning the time to exhaustion test. The trainer then set the treadmill (Spirit Fitness ST-550, Jonesboro, AR) to 4.5 km/h with a 0% inclination and started a stopwatch. After 3 min, the incline was adjusted to 2% and then increased by 2% every 2 min until the time was recorded when subjects were no longer able to continue (23, 24).

### Anthropometric measures

The left and right MUAC were measured in all study visits. A measuring tape on a centimeter scale was placed around the flexed biceps at the midpoint between the shoulder and the tip of the elbow to measure the MUAC.

The body composition was measured using dual-energy X-ray absorptiometry (DEXA, QDR Explorer™, Hologic, Bedford, MA) at the baseline and end of this study.

### Serum biomarkers

Free testosterone (free-T), cortisol, DHT, and CK levels in serum samples were measured using enzyme-linked immunosorbent assay (ELISA). The assay procedures for measuring free-T, DHT, and cortisol (DRG International, Inc., Springfield, NJ), and CK (LifeSpan Biosciences, Inc., Seattle, WA) followed the protocols provided by the vendors. Each serum sample was tested in duplicate. The ELISAs were based on the principles of sandwich immuno-enzymatic reactions. The supplied substrate solutions developed the color reactions, and a microplate reader (Bio-Rad Laboratories, Hercules, CA) recorded the absorbance. The analyte concentrations in the serum samples were calculated from the standard curves plotted in each assay. The sensitivities of the free-T, cortisol, DHT, and CK assay kits were 0.06 pg/ml, 2.5 ng/ml, 6.0 pg/ml, and 0.94 ng/ml, respectively.

A colorimetric assay kit (BioAssay Systems, Hayward, CA) measured the serum LDH activity. The assay kit utilized the MTT tetrazolium salt reduction principle in an NADH-coupled enzymatic reaction, where the reduced form of MTT exhibited an absorption maximum at 565 nm. The intensity of the purple color is directly proportional to the LDH activity. The developed color was recorded using a microplate reader (Bio-Rad Laboratories, Hercules, CA). The enzyme activities in the serum samples were measured by comparing the absorbance values with the standard calibration curve. The assay sensitivity was 2 mU/ml.

## Safety measures

### Hematology and clinical biochemistry evaluations

Serum biochemistry parameters included albumin, alkaline phosphatase, total bilirubin, cholesterol, creatinine, creatine kinase-n-acetyl cysteine, glucose, high-density lipoprotein, low-density lipoprotein, potassium, serum glutamic oxaloacetate transaminase, serum glutamate pyruvate transaminase, triglycerides, and urea. Hematology parameters included total count and differential blood count, erythrocyte sedimentation rate (ESR), hemoglobin, platelet count, mean corpuscular volume, and mean corpuscular hemoglobin. The test parameters in urine analyses include specific gravity, pH, albumin, bile salts, bile pigment, glucose, red blood cells, and ketone bodies. Biochemical parameters were measured using COBAS C 311 (Roche Diagnostics, Rotkreuz, Switzerland); hematological parameters were measured using the MINDRAY BC 20 (Shenzhen Mindray Bio-Medical Electronics Co., Ltd, China) auto hematology analyzer. Urinalysis was carried out using SIEMENS MULTISTIX 10 SG Strips and by microscopy of sediment.

## Statistical analysis

The sample size was calculated using a two-sided *t*-test of the size of 0.05%. The mean difference in the primary endpoint between LI12542F6 and placebo was assumed to be 11, with a typical standard deviation (SD) of 10.2, based on an earlier study conducted on resistance-trained males (25). From these assumptions, the total sample size was calculated as 40 subjects (20 per group in a 1:1 ratio) to obtain a power of 90% of meeting the primary objective.

Clinical data were analyzed on an intention-to-treat basis. The primary comparison evaluated mean change from baseline to end of this study using the Student’s paired *t*-test. Mean change in the treatment group vs. placebo at all evaluations was analyzed using covariance (ANCOVA) analysis, utilizing the baseline measure as a covariate.

## Results

All participants completed the study. Overall, the mean ± SD of the ‘subjects’ adherence to supplementation’ in the placebo and LI12542F6 groups was 99.65 ± 0.92% and 99.34 ± 1.07%, respectively, and the ‘subjects’ adherence to training’ in the placebo and LI12542F6 groups was 97.69 ± 2.59% and 97.87 ± 2.51%, respectively. The baseline characteristics are summarized in Table 2. No significant differences were observed between groups at baseline.

**Table 2:** Participants’ baseline characteristics

Parameters	Placebo (*n* = 20)	LI12542F6 (*n* = 20)
Age (years)	23.35 + 4.18	22.80 ± 3.47
Height (cm)	172.65. ± 5.14	172.83. ± 6.17
Weight (kg)	72.19 ± 13.73	72.23 ± 10.63
BMI (kg/m^2^)	24.16 ± 4.18	24.23 ± 3.73

Data present the mean ± SD.

### Muscle strength

Significant increases in muscle strength were observed for leg and bench press, and grip strength and are summarized in Table 3.

**Table 3:** Effect of LI12542F6 supplementation on 1-RM strength and handgrip strength

Parameters	Evaluations	Placebo (*n* = 20)	LI12542F6 (*n* = 20)
1-RM Bench press (kg)	Baseline	44.45 ± 7.65	42.90 ± 8.39
	Day 14	44.60 ± 8.57	49.40 ± 11.01*^#^
	Day 28	46.10 ± 9.41	66.40 ± 9.29*^#^
	Day 56	49.40 ± 9.54*	70.50 ± 9.30*^#^
	Change from baseline	4.95 ± 2.52	27.60 ± 11.08^#^
1-RM Leg press (kg)	Baseline	58.40 ± 9.17	61.45 ± 13.29
	Day 14	59.55 ± 10.59	69.95 ± 13.91*^#^
	Day 28	60.25 ± 11.40	80.80 ± 13.72*^#^
	Day 56	64.10 ± 10.65*	90.90 ± 11.91*^#^
	Change from baseline	5.70 ± 4.21	29.45 ± 8.29^#^
Handgrip strength (kg)	Baseline	31.50 ± 4.01	32.50 ± 3.44
	Day 14	35.50 ± 3.59*	37.75 ± 3.43*^#^
	Day 28	37.75 ± 4.44*	41.10 ± 3.24*^#^
	Day 56	42.10 ± 4.27*	46.10 ± 3.24*^#^
	Change from baseline	10.60 ± 3.38	13.60 ± 2.46^#^

Data present the mean ± SD.

* indicates significance (*P* < 0.05) in intragroup comparison (vs. baseline) analyzed using student *t*-test;

# indicates significance (*P* < 0.05) in between-the-groups comparison (vs. placebo) analyzed using ANCOVA model with treatment as fixed effects and baseline as a covariate.

### Bench press

After 14 days of supplementation, the 1-RM bench press was increased by 6.50 kg in the LI12542F6-supplemented group (*P* < 0.0001) as compared to the placebo group (0.15 kg; *P* = 0.9909). At the end of this study, the muscle strength of the LI12542F6 supplemented group increased by 27.60 kg (*P* < 0.0001), while the placebo showed a 4.95 kg (*P* = 0.0049) increase from baseline. Also, the intergroup analysis showed that the LI12542F6-supplemented participants exhibited significantly greater gains in strength than the placebo (Table 3).

### Leg press

Leg strength, as assessed by 1-RM leg press, increased from baseline at 14 days by 8.50 kg in the LI12542F6 group (*P* < 0.0001), while the placebo increased by 1.15 kg (*P* = 0.1624). The differences in strength relative to baseline were statistically significant between groups (*P* < 0.0001). On day 56, the 1-RM strength in leg press increased in both LI12542F6-supplemented and placebo groups (29.45 kg, *P* < 0.0001 and 5.70 kg, *P* = 0.0005, respectively) from baseline. Also, in comparison with the placebo, LI12542F6 participants achieved a greater increase (*P* < 0.0001) in leg muscle strength at the end of the intervention (Table 3).

### Handgrip strength

Increases in handgrip strength were statistically significant from baseline in both groups, starting from day 14 through the end of the trial. However, the subjects in the LI12542F6 group showed greater gains; a statistically significant between-group difference was noted at 14 days of treatment (*P* = 0.0079 vs. placebo). The between-group comparison analysis shows the improvement in the LI12542F6 group after 56 days of supplementation (*P* = 0.0006 vs. placebo) (Table 3).

### Secondary outcomes

Endurance outcomes, assessed as the number of cable pull-down repetitions and time to exhaustion on a treadmill (Table 4), were significantly improved at the end of this study. In both groups, increases in the number of repetitions of cable pull-down exercises were significantly increased compared to the baseline from day 14 and gradually increased until the end of this study. Compared to the placebo, the LI12542F6-supplemented participants exhibited significantly greater gains in the number of repetitions throughout the study (Table 4).

**Table 4:** Effect of LI12542F6 supplementation on the endurance of the participants

Parameters	Evaluations	Placebo (*n* = 20)	LI12542F6 (*n* = 20)
Number of repetitions	Baseline	3.40 ± 1.88	3.15 ± 1.27
	Day 14	4.15 ± 1.95*	5.45 ± 1.47*^#^
	Day 28	4.70 ± 2.49*	6.95 ± 1.39*^#^
	Day 56	6.00 ± 2.58*	8.25 ± 1.45*^#^
	Change from baseline	2.60 ± 1.60	5.10 ± 1.29^#^
Time to exhaust (min)	Baseline	22.43 ± 6.13	19.35 ± 5.92
	Day 14	22.45 ± 5.80	20.38 ± 5.06*^#^
	Day 28	22.69 ± 4.61*	21.73 ± 5.14*^#^
	Day 56	24.75 ± 4.24*	24.17 ± 4.92*^#^
	Change from baseline	2.32 ± 2.64	4.82 ± 1.49^#^

Data present the mean ± SD.

* indicates significance (*P* < 0.05) in intragroup comparison (vs. baseline) analyzed using student t-test;

# indicates significance (*P* < 0.05) in between-the-groups comparison (vs. placebo) analyzed using ANCOVA model with treatment as fixed effects and baseline as a covariate.

On day 14 of the study, time to exhaustion in the LI12542F6 and placebo groups was increased by 1.03 ± 1.05 min (*P* < 0.0001) and 0.02 ± 0.76 min (*P* = 0.2528), respectively, from baseline. At the end of this study, the LI12542F6 group showed significantly increased time to exhaustion compared to the placebo (*P* = 0.0008) as well as baseline (*P* < 0.0001) (Table 4).

MUAC was measured for both right and left arms at baseline and on day 56; data are summarized in Table 5. Both left and right MUAC increased in the LI12542F6 group. These increases were statistically significant relative to the baseline (*P* < 0.0001 and *P* = 0.0002, respectively). On day 56, the changes (from baseline) in left and right MUAC were also statistically significant (*P* = 0.0429 and *P* = 0.0427, respectively) as compared to the changes (from baseline) in placebo.

**Table 5:** Effect of LI12542F6 supplementation on Mid-Upper Arm Circumference (MUAC) and Body Composition of the participants

Parameters	Evaluations	Placebo (*n* = 20)	LI12542F6 (*n* = 20)
Left MUAC (cm)	Baseline	33.20 ± 3.40	33.88 ± 2.38
	Day 56	33.34 ± 3.45	34.35 ± 2.57*^#^
Change from baseline (cm)		0.15 ± 0.29	0.48 ± 0.60^#^
Right MUAC (cm)	Baseline	33.73 ± 3.46	34.60 ± 2.26
	Day 56	33.84 ± 3.54	35.05 ± 2.48*^#^
Change from baseline (cm)		0.11 ± 0.26	0.45 ± 0.60^#^
Lean body mass (kg)	Baseline	52.39 ± 7.55	51.39 ± 6.27
	Day 56	52.41 ± 7.92	52.83 ± 6.25*^#^
Change from baseline (kg)		0.02 ± 2.45	1.44 ± 1.58^#^
Total body fat (kg)	Baseline	17.46 ± 7.08	18.48 ± 6.47
	Day 56	17.26 ± 7.15	17.51 ± 5.92*^#^
Change from baseline (kg)		-0.20 ± 0.42	-0.97 ± 1.45^#^

Data present the mean ± SD.

* indicates significance (*P* < 0.05) in intragroup comparison (vs. baseline) analyzed using student *t*-test;

# indicates significance (*P* < 0.05) in between-the-groups comparison (vs. placebo) analyzed using ANCOVA model with treatment as fixed effects and baseline as a covariate.

DEXA analyses exhibited significant changes in body composition on day 56 compared to the baseline. A mean increase of 1.44 kg (*P* = 0.0040) lean mass and a mean reduction of 0.97 kg (*P* = 0.0002) fat mass in the LI12542F6-supplemented subjects were observed (Table 5). Also, on day 56, the changes (from baseline) in lean and fat mass were statistically significant (*P* = 0.0410 and *P* = 0.0338, respectively) relative to the changes (from baseline) in placebo.

### Serum biomarkers

In serum, Free-T, DHT, and cortisol levels were measured at baseline and on day 56; data are shown in Table 6. Free-T was significantly increased in the LI12542F6 group (*P* = 0.0105) from baseline, while a slight reduction was observed in the placebo group. The change from baseline values represents a statistically significant between-group difference (*P* = 0.0128).

**Table 6:** Effect of LI12542F6 supplementation on serum hormone levels of the participants

Parameters	Groups	Baseline	Day 56
Free-testosterone (pg/ml)	Placebo (*n* = 19)	18.49 ± 6.13	17.36 ± 6.03
	LI12542F6 (*n* = 19)	18.80 ± 6.04	21.96 ± 6.08*^#^
Dihydrotestosterone (pg/ml)	Placebo (*n* = 20)	697.51 ± 250.56	835.36 ± 285.90
	LI12542F6 (*n* = 20)	694.54 ± 173.92	891.51 ± 402.27*
Cortisol (ng/ml)	Placebo (*n* = 17)	191.66 ± 56.30	185.16 ± 70.43
	LI12542F6 (*n* = 17)	175.09 ± 49.751	138.13 ± 47.43*^#^

Data present the mean ± SD.

* indicates significance (*P* < 0.05) in intragroup comparison (vs. baseline) analyzed using student *t*-test;

# indicates significance (*P* < 0.05) in between-the-groups comparison (vs. placebo) analyzed using ANCOVA model with treatment as fixed effects and baseline as a covariate.

DHT is an endogenous androgenic sex hormone. DHT levels were significantly increased from baseline in the LI12542F6 group (*P* = 0.0096). The difference between groups did not reach statistical significance.

Cortisol levels were significantly decreased from baseline in the LI12542F6 group (*P* = 0.0057). The change from baseline in placebo was not statistically significant (*P* = 0.9782). The change in cortisol levels from baseline represents a statistically significant between-group difference (*P* = 0.0469).

No significant changes were observed in serum levels of CK and LDH in intra- and intergroup analyses (data not shown).

### Safety outcomes

Safety parameters, including AEs, vital signs, and physical examination, were assessed at all the visits. No AEs were reported during the study. Analyses of vital signs, clinical chemistry, and hematology values were within the normal ranges. The observations on hematology parameters and the clinical chemistry values in the serum samples of the participants at the screening visit and the end of this study are presented in Table 7.

**Table 7:** Effect of LI12542F6 supplementation on the hematology and serum clinical chemistry parameters of the participants

	Parameters	Evaluations	Placebo (*n* = 20)	LI12542F6 (*n* = 20)
Hematology	Hemoglobin (g/dL)	Screening	14.52 ± 0.82	14.66 ± 0.94
		Day 56	14.69 ± 0.96	14.74 ± 0.85
	Platelet count (10^5^/μL)	Screening	2.70 ± 0.64	2.33 ± 0.57
		Day 56	2.69 ± 0.59	2.55 ± 0.41
	Red blood cell (10^6^/μL)	Screening	5.59 ± 0.75	5.38 ± 0.35
		Day 56	5.39 ± 0.37	5.18 ± 0.36
	Total white blood cells (/μL)	Screening	7,040 ± 1,377	7,580 ± 1,278
		Day 56	7,520 ± 1,586	7,345 ± 1,414
	Neutrophils (%)	Screening	61.95 ± 6.43	62.95 ± 7.00
		Day 56	69.0 5 ± 6.21*	62.40 ± 5.94^#^
	Lymphocytes (%)	Screening	31.45 ± 5.97	30.10 ± 6.50
		Day 56	24.85 ± 4.79*	31.60 ± 5.76^#^
	Eosinophil (%)	Screening	2.8 5 ± 1.81	3.20 ± 1.94
		Day 56	3.50 ± 2.48	2.80 ± 1.20
	Monocytes (%)	Screening	3.80 ± 0.52	3.75 ± 0.55
		Day 56	3.00 ± 1.38*	3.15 ± 1.04*
	Basophils (%)	Screening	0.00 ± 0.00	0.00 ± 0.00
		Day 56	0.00 ± 0.00	0.05 ± 0.22
Serum clinical chemistry	Fasting glucose (mg/dL)	Screening	73.75 ± 7.75	78.55 ± 7.34
		Day 56	88.25 ± 7.98*	88.40 ± 8.41*
	Creatinine (mg/dL)	Screening	0.76 ± 0.14	0.77 ± 0.12
		Day 56	0.85 ± 0.11*	0.89 ± 0.11*
	Blood urea nitrogen (mg/dL)	Screening	10.10 ± 2.88	10.20 ± 2.76
		Day 56	10.40 ± 2.72	11.05 ± 2.87
	Bilirubin (mg/dL)	Screening	0.65 ± 0.34	0.58 ± 0.17
		Day 56	0.70 ± 0.28	0.66 ± 0.23
	Aspartate transaminase (U/L)	Screening	29.05 ± 7.20	27.50 ± 8.82
		Day 56	27.35 ± 6.96	26.00 ± 5.86
	Alanine transaminase (U/L)	Screening	27.75 ± 6.38	29.85 ± 11.73
		Day 56	28.45 ± 10.22	25.55 ± 8.70*
	Alkaline phosphate (IU/L)	Screening	85.90 ± 16.68	86.25 ± 20.90
		Day 56	91.20 ± 17.29	86.30 ± 16.57
	Sodium (mEq/L)	Screening	139.60 ± 2.46	139.15 ± 1.09
		Day 56	140.15 ± 2.16	139.30 ± 1.75
	Potassium (mEq/L)	Screening	4.22 ± 0.25	4.22 ± 0.18
		Day 56	4.26 ± 0.22	4.23 ± 0.23
	Albumin (g/dL)	Screening	4.33 ± 0.24	4.35 ± 0.17
		Day 56	4.42 ± 0.19	4.46 ± 0.25
	Low-density lipoprotein (mg/dL)	Screening	82.00 ± 21.68	86.90 ± 29.54
		Day 56	85.80 ± 21.90	89.20 ± 24.90
	High-density lipoprotein (mg/dL)	Screening	37.90 ± 7.95	42.85 ± 7.27^#^
		Day 56	39.80 ± 9.56*	41.55 ± 7.13
	Very low-density lipoprotein (mg/dL)	Screening	23.40 ± 11.00	21.95 ± 9.56
		Day 56	27.00 ± 13.67	27.00 ± 12.75*
	Triglyceride (mg/dL)	Screening	118.30 ± 54.32	111.30 ± 47.68
		Day 56	133.50 ± 64.84	135.75 ± 64.32*
	Total cholesterol (mg/dL)	Screening	143.30 ± 24.88	151.70 ± 35.23
		Day 56	152.60 ± 25.65*	157.75 ± 30.89

Data present the mean ± SD.

* and

# indicate significance (*P* < 0.05) in intragroup comparison (vs. baseline) and between-the-groups comparison (vs. placebo), respectively, analyzed using student *t*-test.

## Discussion

The present randomized, double-blind clinical trial shows encouraging observations as a proof-of-concept on a proprietary botanical formulation, LI12542F6, to improve muscle strength, muscle size, and endurance performance in young men participating in resistance training. Improvements in efficacy measures of the LI12542F6 supplemented subjects were consistent from day 14 through the end of this study.

In this study, LI12542F6-supplemented subjects exhibited substantial increases in 1-RM strength in bench and leg press relative to baseline and placebo. These observations are comparable with earlier investigations conducted on other nutraceutical ingredients. Wankhede et al. demonstrated that 8 weeks of supplementation of *Withania somnifera* extract (600 mg/day) increased 1-RM strength in bench and leg press in young adults (26). Silva et al. demonstrated that daily supplementation of 3 g hydroxymethyl butyrate (HMB) increased strength in young, healthy adults following a resistance training protocol (9). Creatine supplementation at doses ranging from 3 to 30 g/day has also shown diverse effects on physical activity in athletes and non-athletes, including postexercise recovery and protection from exercise-induced muscle damage (27). In this study, the strength benefits achieved through LI12542F6 supplementation suggest that this botanical formulation is an exciting addition to the repertoire available to physically active individuals interested in enhancing muscle mass, strength, and exercise performances.

In addition to increased muscle strength shown in this study, LI12542F6 supplementation improved other endpoints for muscle and endurance. Following 56 days of supplementation, the number of repetitions by cable pull-down was increased 1.96-fold in the LI12542F6 group compared to the placebo group. In addition, time to exhaustion on the treadmill was also increased by 24.91% in the LI12542F6 group compared to 10.34% in the placebo group.

Our unpublished observations indicated that LI12542F6 potentially increased mitochondrial function in skeletal muscle cells via eNOS signaling *in vitro* (unpublished observations). Based on these observations, we postulated that this unique herbal blend would enhance muscle growth, strength, and exercise performance in males. Enhanced eNOS activity improves mitochondrial function and increases the generation of new mitochondria through PGC1α upregulation to enhance oxygen utilization in the muscle cells, which is critical for improved muscle performance (10, 11). Based on scientific understanding, we believe that the mode of action of this novel botanical formulation is unlike that of conventional NO boosters. The ergogenic benefits of these NO boosters, such as L-arginine, citrulline, or dietary nitrates, are transient (28, 29). There is insufficient evidence supporting these NO boosters on eNOS activation and improved mitochondrial function. Recently, Barros et al. have shown that L-arginine treatment increases NO production but does not alter the mitochondrial function in human osteosarcoma cells *in vitro* (30).

Prolonged and strenuous exercise increases reactive oxygen species (ROS) and free radicals, adversely impacting normal muscle physiology, perturbing redox balance, and reducing endurance performance (31). Extracts of *S. indicus* (13) and *M. indica* bark (32) are potent antioxidants that scavenge intracellular free radicals. Our observation of elevated endurance levels in the LI12542F6 supplemented participants suggests the possibility that the free radical scavenging potential of the herbal formulation may support additional beneficial effects. Further investigations are needed to explore the precise mechanisms of action.

While biomarkers of muscle metabolism were not analyzed in this initial study, there are several other potential mechanisms through which LI12542F6 may exert its effects. For example, muscle response to resistance training involves increased protein synthesis via transcriptional and translational mechanisms, perturbation of cellular homeostasis, and increased muscle cell growth (33). As mentioned in the “Introduction” section, the development of LI12542F6 involved *in vitro* screens of pathways known to be involved in muscle metabolism. During these screens, it was noted that the expression of both the mammalian target of rapamycin (mTOR, serine/threonine protein kinase) and phosphorylated p70 kinase (p70s6k) was increased (unpublished observations). Both proteins/transcription factors are thought to promote muscle hypertrophy in resistance training (33, 34).

Resistance training promotes muscle growth and improves body composition (35). The greater improvements in lean body mass and MUAC measurements in LI12542F6 supplemented participants were observed compared to the placebo, suggesting an anabolic effect of the herbal supplement. Furthermore, this observation is supported by an increased free testosterone level and a decreased cortisol level in the active group. Testosterone (T) is an anabolic hormone; it increases muscle mass, strength, and endurance; in contrast, cortisol produces catabolic effects (36). In this study, we measured free-T levels in the participants. The free form of T binds to androgen receptors and serves as a transcription factor for protein synthesis in the muscles (37). Overall, the effect of LI12542F6 supplementation on the metabolic hormones that regulate muscle growth and performance is encouraging.

The current trial has limitations. In this proof-of-concept study, we evaluated the effect of this botanical formulation on the subjects while maintaining their regular diet. This is a key limitation of the study, given that we did not control the potential impact of calorie and protein consumption. Next, given that greater aerobic capacity is critical for improved endurance performance, it would have been interesting to assess further the effect of LI12542F6 supplementation on aerobic capacity in the present study. Finally, this study evaluated benefits in men during resistance training; research on potential strength and endurance benefits for women is also needed. Our subsequent investigations will plan to address these gaps.

The data from the safety parameters assessments and the AEs record suggest that LI12542F6 was well-tolerated by the study participants. In agreement with broad-spectrum preclinical toxicity study data (19), the present observations further support the safety and tolerability of LI12542F6. Together, the observations from this study on increased muscle growth, strength, and enhanced endurance in LI12542F6-supplemented participants support our hypothesis.

## Conclusion

This 56-day clinical trial demonstrates that a proprietary herbal blend, LI12542F6 (MyoTOR® or RipFACTOR®), supplementation increased muscle strength, growth, and endurance in young male subjects with resistance training. LI12542F6 supplementation is also efficacious in improving lean body mass and modulating free testosterone and cortisol that influence muscle protein metabolism. Importantly, this herbal blend is well-tolerated by the participants.

## References

[1] Volaklis KA, Halle M, Meisinger C. Muscular strength as a strong predictor of mortality: a narrative review. Eur J Intern Med 2015; 26(5): 303–10. doi: 10.1016/j.ejim.2015.04.013 25921473

[2] Valenzuela PL, Morales JS, Emanuele E, Pareja-Galeano H, Lucia A. Supplements with purported effects on muscle mass and strength. Eur J Nutr 2019; 58(8): 2983–3008. doi: 10.1007/s00394-018-1882-z 30604177

[3] Morton RW, Murphy KT, McKellar SR, Schoenfeld BJ, Henselmans M, Helms E, et al. A systematic review, meta-analysis and meta-regression of the effect of protein supplementation on resistance training-induced gains in muscle mass and strength in healthy adults. Br J Sports Med 2018; 52(6): 376–84. doi: 10.1136/bjsports-2017-097608 28698222 PMC5867436

[4] Stokes T, Hector AJ, Morton RW, McGlory C, Phillips SM. Recent perspectives regarding the role of dietary protein for the promotion of muscle hypertrophy with resistance exercise training. Nutrients 2018; 10(2): 180. doi:10.3390/nu10020180. 29414855 PMC5852756

[5] Affourtit C, Bailey SJ, Jones AM, Smallwood MJ, Winyard PG. On the mechanism by which dietary nitrate improves human skeletal muscle function. Front Physiol 2015; 6: 211. doi: 10.3389/fphys.2015.00211 26283970 PMC4518145

[6] McLellan TM, Caldwell JA, Lieberman HR. A review of caffeine’s effects on cognitive, physical and occupational performance. Neurosci Biobehav Rev 2016; 71: 294–312. doi: 10.1016/j.neubiorev.2016.09.001 27612937

[7] Deane CS, Wilkinson DJ, Phillips BE, Smith K, Etheridge T, Atherton PJ. ‘Nutraceuticals’ in relation to human skeletal muscle and exercise. Am J Physiol Endocrinol Metab 2017; 312(4): E282–99. doi: 10.1152/ajpendo.00230.2016 28143855 PMC5406990

[8] Asadi A, Arazi H, Suzuki K. Effects of beta-Hydroxy-beta-methylbutyrate-free acid supplementation on strength, power and hormonal adaptations following resistance training. Nutrients 2017; 9(12): 1316. doi: 10.3390/nu9121316 29207472 PMC5748766

[9] Silva VR, Belozo FL, Micheletti TO, Conrado M, Stout JR, Pimentel GD, et al. β-hydroxy-β-methylbutyrate free acid supplementation may improve recovery and muscle adaptations after resistance training: a systematic review. Nutr Res 2017; 45: 1–9. doi: 10.1016/j.nutres.2017.07.008 29037326

[10] Dyakova EY, Kapilevich LV, Shylko VG, Popov SV, Anfinogenova Y. Physical exercise associated with NO production: signaling pathways and significance in health and disease. Front Cell Dev Biol 2015; 3: 19. doi: 10.3389/fcell.2015.00019 25883934 PMC4382985

[11] Slizik M, Pospieszna B, Gronek J, Sworek R. Are SNIP’s still desirable in sports genomics? Trends Sport Sci 2017; 1(24): 13–18.

[12] Groennebaek T, Vissing K. Impact of resistance training on skeletal muscle mitochondrial biogenesis, content, and function. Front Physiol 2017; 8: 713. doi: 10.3389/fphys.2017.00713 28966596 PMC5605648

[13] Ramachandran S. Review on *Sphaeranthus indicus* Linn. (Kottaikkarantai). Pharmacogn Rev 2013; 7(14): 157–69. doi: 10.4103/0973-7847.120517 24347924 PMC3841994

[14] George M, Joseph L, Sujith K, Paul NM. *Sphaeranthus indicus* Linn: a pharmacological update. Pharm Innov 2017: 6(2): 77–84.

[15] Galani VJ, Patel BG, Rana DG. *Sphaeranthus indicus* Linn.: a phytopharmacological review. Int J Ayurveda Res 2010; 1(4): 247–53. doi: 10.4103/0974-7788.76790 21455454 PMC3059449

[16] Batool N, Ilyas N, Shabir S, Saeed M, Mazhar R, Amjid MW. Mini-review – a mini-review of therapeutic potential of *Mangifera indica* L. Pak J Pharm Sci 2018; 31(4): 1441–8. 30033432

[17] Yoshimi N, Matsunaga K, Katayama M, Yamada Y, Kuno T, Qiao Z, et al. The inhibitory effects of mangiferin, a naturally occurring glucosylxanthone, in bowel carcinogenesis of male F344 rats. Cancer Lett 2001; 163(2): 163–70. doi: 10.1016/s0304-3835(00)00678-9 11165750

[18] Prado Y, Merino N, Acosta J, Herrera JA, Luque Y, Hernández I, et al. Acute and 28-day subchronic toxicity studies of mangiferin, a glucosyl xanthone isolated from *Mangifera indica* L. stem bark. J Pharm Pharmacogn Res 2015; 3(1): 13–23.

[19] Nestmann ER, Alluri VK, Dodda S, Davis BA. Toxicological studies on the botanical supplement LI12542F6 containing extracts of *Sphaeranthus indicus* flower heads and *Mangifera indica* (mango tree) bark. Food Sci Nutr 2019; 7(2): 817–33. doi: 10.1002/fsn3.931 30847161 PMC6392882

[20] Baechle T, Earle R, Wathen M. Resistance training. In: Baechle T, Earle R, eds. Essentials of strength training and conditioning. 3rd ed. Champaign, IL: Human Kinetics; 2008, pp. 381–411.

[21] Centers for Disease Control and Prevention. National Health and Nutrition Examination Survey (NHANES): muscle strength procedures manual. Atlanta, GA; 2011. Available from: https://wwwn.cdc.gov/nchs/data/nhanes/2011-2012/manuals/Muscle_Strength_Proc_Manual.pdf [cited 24 May 2016].

[22] Signorile JF, Zink AJ, Szwed SP. A comparative electromyographical investigation of muscle utilization patterns using various hand positions during the lateral pull-down. J Strength Cond Res 2002; 16(4): 539–46. 12423182

[23] Crowther RG, Leicht AS, Spinks WL, Sangla K, Quigley F, Golledge J. Effects of a 6-month exercise program pilot study on walking economy, peak physiological characteristics, and walking performance in patients with peripheral arterial disease. Vasc Health Risk Manag 2012; 8: 225–32. doi: 10.2147/VHRM.S30056 22566743 PMC3346266

[24] Pal MS, Majumdar D, Pramanik A, Chowdhury B, Majumdar D. Optimum load for carriage by Indian soldiers on different uphill gradients at specified walking speed. Int J Ind Ergon 2014; 44(2): 260–5. doi: 10.1016/j.ergon.2013.09.001

[25] Konda MR, Alluri KV, Janardhanan PK, Golakoti T. Sengupta K. Combined extracts of Garcinia mangostana fruit rind and Cinnamomum tamala leaf supplementation enhances muscle strength and endurance in resistance trained males. J Int Soc Sports Nutr 2018; 15: 50. doi: 10.1186/s12970-018-0257-4 30348185 PMC6196563

[26] Wankhede S, Langade D, Joshi K, Sinha SR, Bhattacharyya S. Examining the effect of Withania somnifera supplementation on muscle strength and recovery: a randomized controlled trial. J Int Soc Sports Nutr 2015; 12: 43. doi: 10.1186/s12970-015-0104-9 26609282 PMC4658772

[27] Kreider RB, Kalman DS, Antonio J, Ziegenfuss TN, Wildman R, Collins R, et al. International Society of Sports Nutrition position stand: safety and efficacy of creatine supplementation in exercise, sport, and medicine. J Int Soc Sports Nutr 2017; 14: 18. doi: 10.1186/s12970-017-0173-z 28615996 PMC5469049

[28] Bescos R, Sureda A, Tur JA, Pons A. The effect of nitric-oxide-related supplements on human performance. Sports Med 2012; 42(2): 99–117. doi: 10.2165/11596860-000000000-0000 22260513

[29] Bernardo DN, Bryk FF, Fucs PM. Influence of nitric oxide in the improvement of muscle power. Acta Ortop Bras 2015; 23(6): 294–8. doi: 10.1590/1413-785220152306148249 27057140 PMC4775504

[30] Barros CDS, Livramento JB, Mouro MG, Higa EMS, Moraes CT, Tengan CH. L-Arginine reduces nitro-oxidative stress in cultured cells with mitochondrial deficiency. Nutrients 2021; 13(2): 534. doi: 10.3390/nu13020534 33562042 PMC7914615

[31] Kawamura T, Muraoka I. Exercise-induced oxidative stress and the effects of antioxidant intake from a physiological viewpoint. Antioxidants 2018; 7(9): 119. doi: 10.3390/antiox7090119 30189660 PMC6162669

[32] Pardo-Andreu GL, Barrios MF, Curti C, Hernandez I, Merino N, Lemus Y, et al. Protective effects of Mangifera indica L extract (Vimang), and its major component mangiferin, on iron-induced oxidative damage to rat serum and liver. Pharmacol Res 2008; 57(1): 79–86. doi: 10.1016/j.phrs.2007.12.004 18243014

[33] Coffey VG, Hawley JA. The molecular basis of training adaptation. Sports Med 2007; 37(9): 737–63. doi: 10.2165/00007256-200737090-00001 17722947

[34] Camera DM, Smiles WJ, Hawley JA. Exercise-induced skeletal muscle signaling pathways and human athletic performance. Free Radic Biol Med 2016; 98: 131–43. doi: 10.1016/j.freeradbiomed.2016.02.007 26876650

[35] Benito PJ, Cupeiro R, Ramos-Campo DJ, Alcaraz PE, Rubio-Arias JA. A systematic review with meta-analysis of the effect of resistance training on whole-body muscle growth in healthy adult males. Int J Environ Res Public Health 2020; 17(4): 1285. doi: 10.3390/ijerph17041285 32079265 PMC7068252

[36] Keizer H, Janssen GM, Menheere P, Kranenburg G. Changes in basal plasma testosterone, cortisol, and dehydroepiandrosterone sulfate in previously untrained males and females preparing for a marathon. Int J Sports Med 1989; 10: S139–45. doi: 10.1055/s-2007-1024962 2532181

[37] Kraemer WJ, Ratamess NA, Hymer WC, Nindl BC, Fragala MS. Growth hormone(s), testosterone, insulin-like growth factors, and cortisol: roles and integration for cellular development and growth with exercise. Front Endocrinol 2020; 11: 33. doi: 10.3389/fendo.2020.00033 PMC7052063 32158429

